# Psychosomatic and Psychological Impact of Hepatitis C: A Cross-Sectional Study of Depression, Fibrosis, and Somatic Symptoms in Treatment-Naive and Treatment-Experienced Patients Who Achieved Sustained Virologic Response

**DOI:** 10.7759/cureus.80104

**Published:** 2025-03-05

**Authors:** Ahmad A Rashid, Akhtar Rashid, Muqadas Zahra, Noman Aslam, Safa Jameel, Hafiz M Awais, Hamas Zaheer

**Affiliations:** 1 Medicine and Surgery, Sargodha Medical College, Sargodha, PAK; 2 Internal Medicine, Al-Rashid Hospital, Sargodha, PAK; 3 Gastroenterology, Dr. Faisal Masood Teaching Hospital, Sargodha, PAK; 4 Medicine, Services Hospital Lahore, Lahore, PAK

**Keywords:** depression in chronic illness, direct-acting antiviral, fibrosis-4 (fib-4) score, hepatitis c (hcv), psychosomatic symptoms

## Abstract

Background: Hepatitis C virus (HCV) infection is associated with neuropsychiatric symptoms, including depression and psychosomatic complaints. While direct-acting antivirals (DAAs) have revolutionized HCV treatment, their impact on mental health and systemic symptoms remains unclear. This study aimed to compare depression severity, liver fibrosis scores, and psychosomatic symptoms in treatment-naive (Tx-naive) and treatment-experienced (Tx-experienced) patients who achieved sustained virologic response (SVR).

Materials and methods: A cross-sectional observational study was conducted at two tertiary care hospitals in Pakistan. Patients with chronic HCV were categorized into Tx-naive and Tx-experienced SVR-achieved groups. Depression was assessed using the Patient Health Questionnaire-9 (PHQ-9), while fibrosis severity was determined using Fibrosis-4 (FIB-4) scores. Psychosomatic symptoms, including myalgia, asthenia, and functional dyspepsia, were documented. Statistical comparisons were performed using independent t-tests, Mann-Whitney U tests, and Chi-squared tests.

Results: A total of 142 patients were analyzed, with 69 in the Tx-naive group and 73 in the Tx-experienced SVR-achieved group. PHQ-9 scores did not significantly differ between groups (p=0.343) or FIB-4 scores (p=0.691). The prevalence of psychosomatic symptoms was comparable across both cohorts, with no statistically significant differences in individual symptoms such as asthenia, myalgia, or burning feet syndrome (all p>0.05).

Conclusions: The findings suggest that HCV itself, rather than DAA therapy, is the primary contributor to depression and somatic symptoms. Achieving SVR does not significantly alter mental health or systemic symptom burden. These results highlight the need for long-term neuropsychiatric monitoring in HCV survivors, as symptoms may persist despite viral clearance. Future research should explore the biological underpinnings of these persistent complaints and assess potential interventions for improving patient quality of life.

## Introduction

Hepatitis C virus (HCV) is an RNA-enveloped virus in the *Flaviviridae* family and *Hepacivirus* genus transmitted through the blood and blood-derived products. Indeed, until screening blood for transfusions was introduced, it was the most common cause of transfusion-induced hepatitis or post-transfusion hepatitis. According to a recent survey, about 71.1 million people are chronically infected with the HCV [1]. It is important to note that the number of new cases of HCV has shown an increasing trend in recent years, with a notable rise in the past several months. Individuals at risk for developing HCV are healthcare workers because of needlestick and chronic hemodialysis patients and their spouses. Hepatitis C, like in other countries, is also on the rise and is considered an alarming health issue. According to a recent survey, the prevalence of hepatitis C in the general population of Pakistan in the year 2022 was 11.32% [2].

HCV infection typically begins with an acute phase, which is often asymptomatic or presents nonspecific symptoms such as fatigue, malaise, abdominal discomfort, jaundice, and nausea. In the majority of cases, the infection becomes chronic, and over several decades, it can lead to significant liver damage. Pathogenesis involves direct viral cytotoxicity and immune-mediated mechanisms, resulting in chronic inflammation and progressive liver fibrosis [3]. Without treatment, chronic HCV can advance to cirrhosis, liver failure, and hepatocellular carcinoma.

HCV's persistence is due to its ability to evade the host immune response through rapid mutation and suppression of antiviral immune pathways. Early detection and treatment with direct-acting antivirals (DAAs) have significantly improved outcomes, with the potential for complete viral clearance and reduction in liver-related complications.

HCV plays a significant role in the development of depression, neurocognitive deficits, and anemia through its ability to induce chronic inflammation and disrupt various physiological systems. In particular, elevated levels of pro-inflammatory cytokines such as interleukin-1 (IL-1), interleukin-6 (IL-6), and tumor necrosis factor-alpha (TNF-α) are observed in chronic HCV infection. These cytokines can cross the blood-brain barrier and affect neurotransmitter systems, notably serotonin and dopamine pathways, which are crucial in mood regulation. This dysregulation is a key factor in the development of depression, which is prevalent in HCV patients even in the absence of antiviral treatment [4]. Chronic immune activation and inflammatory cytokine release also contribute to cognitive dysfunction, as seen in deficits in attention, memory, and executive function among HCV-infected individuals.

The role of cytokines extends beyond mood and cognition; they are implicated in developing anemia, a common complication in HCV. Inflammatory cytokines like IL-6 can inhibit erythropoiesis by suppressing erythropoietin production and impairing iron metabolism, leading to anemia of chronic disease. This can exacerbate symptoms of fatigue and weakness, contributing further to the psychological and physical burden on the patient. Moreover, anemia, particularly when worsened by HCV treatments such as ribavirin, can compound neurocognitive deficits and depressive symptoms, creating a cycle of worsening mental and physical health [5].

Emerging evidence, such as the observational study conducted by Dan et al., also suggests that HCV may invade the central nervous system and replicate at low levels, causing direct neuroinflammation and further complicating its impact on mental health. The combination of chronic inflammation, cytokine dysregulation, and potential direct viral effects on the brain underscores the multifaceted ways in which HCV contributes to depression, cognitive decline, and systemic complications such as anemia [6]. Anemia in HCV patients can significantly impact overall health, contributing to fatigue, cognitive impairment, and reduced quality of life while also potentially exacerbating preexisting neuropsychiatric symptoms, further complicating disease management.

Different treatment methods have been implicated in the treatment of acute as well as chronic hepatitis C. In the past, INF-α, despite the success of the treatment in clearing the virus, was associated with new-onset depression and neuropsychiatric symptoms. In a study, it is mentioned that the risk for emergent and new-onset depression and neuropsychiatric effects in patients treated with this drug is 30-70% [7]. The precise mechanism for this is unknown; however, it is proposed that interferon stimulates the production of pro-inflammatory cytokines, such as IL-6 and TNF-α, which can cross the blood-brain barrier and alter neurotransmitter systems, particularly those regulating serotonin and dopamine. This neuroinflammatory response disrupts normal mood regulation, leading to depressive symptoms in many patients during therapy [8].

Due to this, a newer class of drugs called DAAs was invented. DAAs currently in clinical medicine include NS5A/NS5B inhibitors, which inhibit the RNA polymerase. Out of these, sofosbuvir/velpatasvir/voxilaprevir are the most efficacious [9]. These drugs were virtually distinct and carried a less adverse effect profile as compared to interferon-based therapy. Still, the effect of DAAs on mood and depressive symptoms has always been a topic of dispute in literature. Much of the literature points toward a "no effect” of DAA therapy on mood and depression, while another points toward a worsening of mood and onset of new depression, and some also point toward improvement of mood after DAA therapy. A study puts the risk of major depressive symptoms and other depression following DAA therapy at 13% and 46.3%, respectively [10]. According to a nationwide cohort study, DAA therapy was associated with a lower risk of neuropsychiatric disorders as compared to the INF group. However, there was still an incidence of depression [11]. According to another study, to the researchers' surprise, DAA therapy did not affect anxiety or depression [12]. The depressive symptoms were also exacerbated by DAA usage in patients with pre-treatment psychiatric symptoms, according to a case report in BMC gastroenterology [13] and a paper published in the European Journal of Hospital Pharmacy [14].

In contrast, the bulk of literature points toward improvement of mood, sleep, and quality of life following DAA therapy in patients [15]. However, some studies also show that DAA therapy renders high cure efficiency (>90%) and a good safety profile, and it may even bring some unexpected benefits to patients [16]. Similar studies are present in numerous pieces of literature.

Despite extensive research on the psychiatric effects of HCV and its treatment, there remains a lack of clarity regarding the role of DAAs in influencing both mental health and systemic symptoms. While some studies have reported the persistence of somatic symptoms such as asthenia, myalgia, and fatigue following both interferon-based and DAA therapy, the definitive role of antiviral treatment in modifying these symptoms remains debated [17,18]. Moreover, there is limited research directly comparing the burden of these psychosomatic symptoms in treatment-naive (Tx-naive) and treatment-experienced (Tx-experienced) patients who achieved sustained virologic response (SVR), making it unclear whether these symptoms are primarily driven by HCV itself, residual inflammation post-SVR, or treatment-related factors.

Additionally, while achieving SVR is known to halt fibrosis progression, the relationship between FIB-4 scores post-SVR and psychological outcomes remains unexplored. Given the conflicting evidence regarding DAAs and their impact on mental and physical well-being, this study aims to provide a more comprehensive understanding by assessing PHQ-9 scores (depression), FIB-4 scores (fibrosis severity), and psychosomatic symptoms (asthenia, myalgia, fatigue, etc.) in both treatment-naive and treatment-experienced SVR-achieved groups.

The objective of this study is to evaluate whether depression persists, improves, or remains unchanged following SVR after DAA therapy in HCV patients. Additionally, the study examines psychosomatic symptoms and the severity of liver fibrosis post-treatment to determine whether these systemic effects follow a similar pattern. By comparing treatment-naive and treatment-experienced SVR-achieved patients, this research aims to provide insights into the broader impact of HCV clearance beyond viral eradication.

## Materials and methods

Study design and setting

This cross-sectional observational study was conducted at two tertiary care Institutions, Dr. Faisal Masood Teaching Hospital Sargodha and Services Institute of Health Sciences Lahore, Punjab, Pakistan, from October 2024 to December 2024. The study aimed to compare depression (PHQ-9), fibrosis (FIB-4), and psychosomatic symptoms (asthenia, myalgia, burning feet, functional dyspepsia, numbness in feet/hands, irritable bowel syndrome (IBS), restless leg syndrome, and cramps) in TX-naive and Tx-experienced SVR-achieved patients with chronic HCV infection. Ethical approval was obtained from the Ethical Review Committee of Sargodha Medical College under IRB ID 2024-1009, and written informed consent was obtained from all participants.

Study population and eligibility criteria

**Figure 1:**
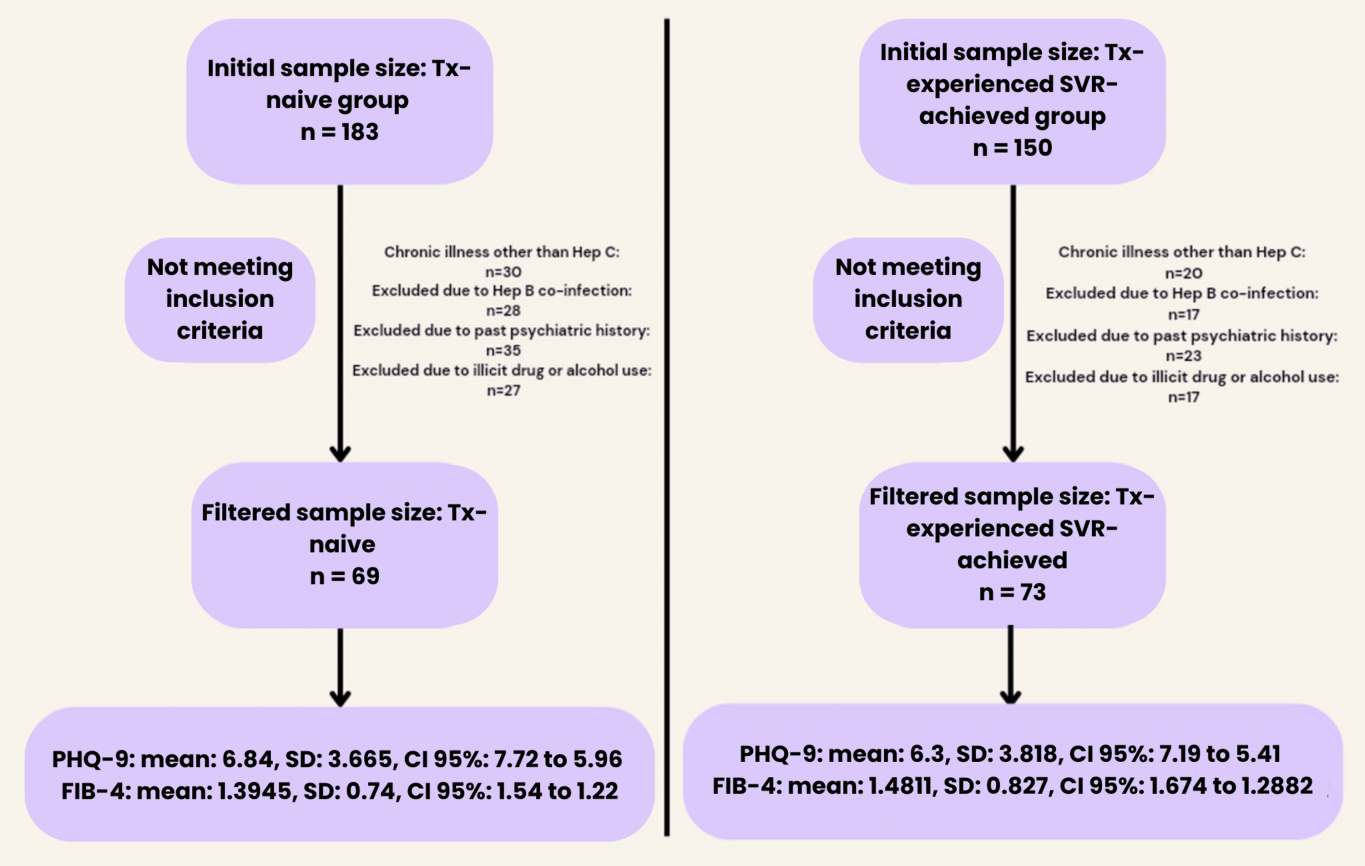
Initial and filtered sample size after excluding the patients not meeting the inclusion criteria Tx: treatment, PHQ-9: Patient Health Questionnaire-9, FIB-4: Fibrosis-4, SD: standard deviation, CI: confidence interval, SVR: sustained virologic response

A total of 183 Tx-naive and 150 Tx-experienced SVR-achieved patients were initially screened. After applying the exclusion criteria, 69 Tx-naive and 73 Tx-experienced SVR-achieved patients were included in the final analysis (Figure 1).

Inclusion Criteria

The inclusion criteria for this study required participants to be over 18 years old with a confirmed diagnosis of chronic HCV infection through polymerase chain reaction (PCR). Patients were categorized into two groups: the Tx-naive group, defined as the patients who have chronic HCV infection confirmed by PCR and have not been treated with DAAs, and the Tx-experienced SVR-achieved group, defined as the group that contains individuals who have undergone a successful treatment period of 12 or 24 weeks by taking DAAs and have achieved SVR, i.e., non-detectable HCV RNA in the blood post 12- or 24-week treatment of HCV by DAAs.

Exclusion Criteria

Exclusion criteria included co-infection with HBV or HIV, as well as a prior history of any chronic illness other than HCV. Patients with a past or current psychiatric illness diagnosed before their HCV diagnosis were also excluded. Evaluation of past or current psychiatric history was done by carefully screening the medical records of the patients and the medications they were taking; patients on antidepressants, antipsychotics, or any other CNS drug that could affect their mood or could act as a confounder were excluded from the study. Additionally, individuals with a history of active or past substance use or alcohol dependence, as well as those with decompensated liver disease or hepatocellular carcinoma, were not eligible for the study. Individuals who spontaneously cleared HCV without treatment were also not included in the study.

Data collection and outcome measures

The data was collected from the patients on well-structured forms, which included the patients’ demographics, including age, sex, marital status, and socioeconomic status. Patients with any of the above-mentioned characteristics of the exclusion criteria were excluded from the study. Depending on whether the patients had a positive diagnosis of hepatitis C (based on PCR or serology) and were not taking any treatment, i.e., the Tx-naive group, or whether they had completed the standard course of 12-week DAA treatment and had achieved SVR, i.e., the Tx-experienced SVR-achieved group, the patients were then included in two different groups: Group A (case group), which included Tx-experienced SVR-achieved patients, and Group B (control group), which included Tx-naive patients. Liver function tests such as alanine transaminase (ALT), aspartate transaminase (AST), and platelet count were also obtained to calculate the FIB-4 scores. To minimize the effect of socioeconomic status as a confounding variable, participants were selected from a relatively homogeneous socioeconomic background. All individuals had access to similar healthcare facilities, educational levels, and living standards, ensuring that socioeconomic disparities did not significantly influence depression scores or other health parameters.

Psychiatric and psychosomatic symptoms were then assessed using validated tools.

Depression Assessment

Depression and its severity were assessed by interviewing the patients according to the PHQ-9, and results in the form of numerical scores were interpreted as mild depression (5-9), moderate depression (10-14), and severe depression ≥15.

The PHQ-9 is a widely used screening tool for evaluating mood disorders, particularly depression and anxiety. The PHQ-9 assesses the severity of depressive symptoms based on the Diagnostic and Statistical Manual of Mental Disorders, 5th Edition (DSM-5) criteria, with high sensitivity and specificity for identifying major depression. It is extensively validated across diverse populations and settings, making it a reliable tool for clinical practice and research. It is a tool favored for its ease of use, cost-effectiveness, and ability to track changes in mood disorders over time, making them indispensable in mental health evaluations [19]. All participants were given the Urdu-translated version of the forms, as it is shown that their accuracy and efficacy in judging depressive and anxiety disorders are comparable to the English version [20]. The English language PHQ-9 form and its Urdu translated version are provided in the Appendices section.

Fibrosis

FIB-4 scores were calculated according to the relevant formula and were interpreted as low fibrosis risk <1.3, intermediate fibrosis risk 1.3-2.67, and high fibrosis risk >2.67.

Psychosomatic Symptoms

Myalgia, asthenia, and other related symptoms were documented based on patient-reported outcomes and recorded as binary variables (yes/no). Psychosomatic symptoms were evaluated by trained physicians and data collectors through specialized patient interviews using a well-designed assessment form. Evaluation of IBS was according to the set criteria of diagnosis, i.e., recurrent abdominal pain, on average, at least one day per week in the last three months, associated with two or more of the following: related to defecation, associated with a change in stool frequency, and associated with a change in stool form (appearance).

Statistical analysis

Data analysis was performed using SPSS Statistics version 29 (IBM Corp. Released 2023. IBM SPSS Statistics for Windows, Version 29.0.2.0 Armonk, NY: IBM Corp.). Continuous variables were reported as mean ± standard deviation (SD) and compared using an independent sample t-test for the normal distribution of PHQ-9 scores and a Mann-Whitney U test for the non-normal distribution of FIB-4 scores. Normality was assessed using both statistical and graphical data. The Kolmogorov-Smirnov test was evaluated to assess deviation from normality. Additionally, quantile-quantile (Q-Q) plots, histograms, and boxplots were inspected to assess data distribution. Skewness and kurtosis were also computed, and values between +1 and -1 were considered normal. A Chi-squared test was used for categorical variables of psychosomatic symptoms, which were recorded as percentages. The statistical significance threshold was set to p<0.05.

## Results

Baseline characteristics

A total of 183 participants from the Tx-naive and 150 participants from the Tx-experienced SVR-achieved group were screened. After further filtering the sample size, a total of 69 participants were included in the Tx-naive group, with 38 females (55.1%) and 31 males (44.9%). A total of 73 participants were included in the Tx-experienced SVR-achieved group, with 54 females (74%) and 19 males (26%). Participants were selected from a controlled socioeconomic background to minimize the influence of financial or healthcare access disparities on study outcomes (Table 1).

**Table 1:** Gender- and age-wise distribution of the participants between the groups Tx-naive: patients not started on DAAs and are diagnosed with chronic HCV infection. Tx-experienced SVR-achieved: patients who have achieved a 12-week course of DAAs. SVR is defined as non-detectable HCV RNA in the blood post 12- or 24-week treatment of HCV by DAAs. Tx: treatment, DAAs: direct-acting antivirals, HCV: hepatitis C virus, RNA: ribonucleic acid, SVR: sustained virologic response

Sex	Tx-naive	Tx-experienced SVR-achieved
Male	31 (44.9%)	19 (26%)
Female	38 (55.1%)	54 (74%)
Mean age ± SD	44 ± 6 years	47 ± 7 years

PHQ-9 scores

The mean PHQ-9 scores in the Tx-naive group were 6.84, with an SD of 3.665 and a 95% CI (5.96 to 7.72). The minimum reported score in this group was a score of zero, and the highest reported score was a score of 17. Among the 69 participants, 36 (52.2%) had mild depression (5-9), 12 (17.4%) had moderate depression (10-14), two (2.9%) had severe depression (>15), and 19 (27.5%) had minimal or no depression (0-4).

The mean PHQ-9 scores in the Tx-experienced SVR-achieved group were 6.30, with an SD of 3.818 and a 95% CI (5.41 to 7.19). The minimum reported score in this group was zero, with a maximum reported score of 16. Among the 73 participants, 33 (45.2%) had mild depression (5-9), 11 (15.1%) had moderate depression (10-14), three (4.1%) had severe depression (>15), and 26 (35.6%) had minimal or no depression (0-4) (Table 2).

**Table 2:** Comparison of PHQ-9 score distribution between Tx-naive and Tx-experienced SVR-achieved groups Tx-naive: patients not started on DAAs and are diagnosed with chronic HCV infection. Tx-experienced SVR-achieved: patients who have achieved a 12-week course of DAAs. SVR is defined as non-detectable HCV RNA in the blood post 12- or 24-week treatment of HCV by DAAs. PHQ-9: Patient Health Questionnaire-9, Tx: treatment, DAAs: direct-acting antivirals, HCV: hepatitis C virus, RNA: ribonucleic acid, SVR: sustained virologic response

PHQ-9 score range	Category	Tx-naive (n=69)	% Tx-naive	Tx-experienced SVR-achieved (n=73)	% Tx-experienced SVR-achieved
0-4	Minimal or no depression	19	27.5%	26	35.6%
5-9	Mild depression	36	52.2%	33	45.2%
10-14	Moderate depression	12	17.4%	11	15.1%
>15	Severe depression	2	2.9%	3	4.1%
Total		69	100%	73	100%

**Figure 2:**
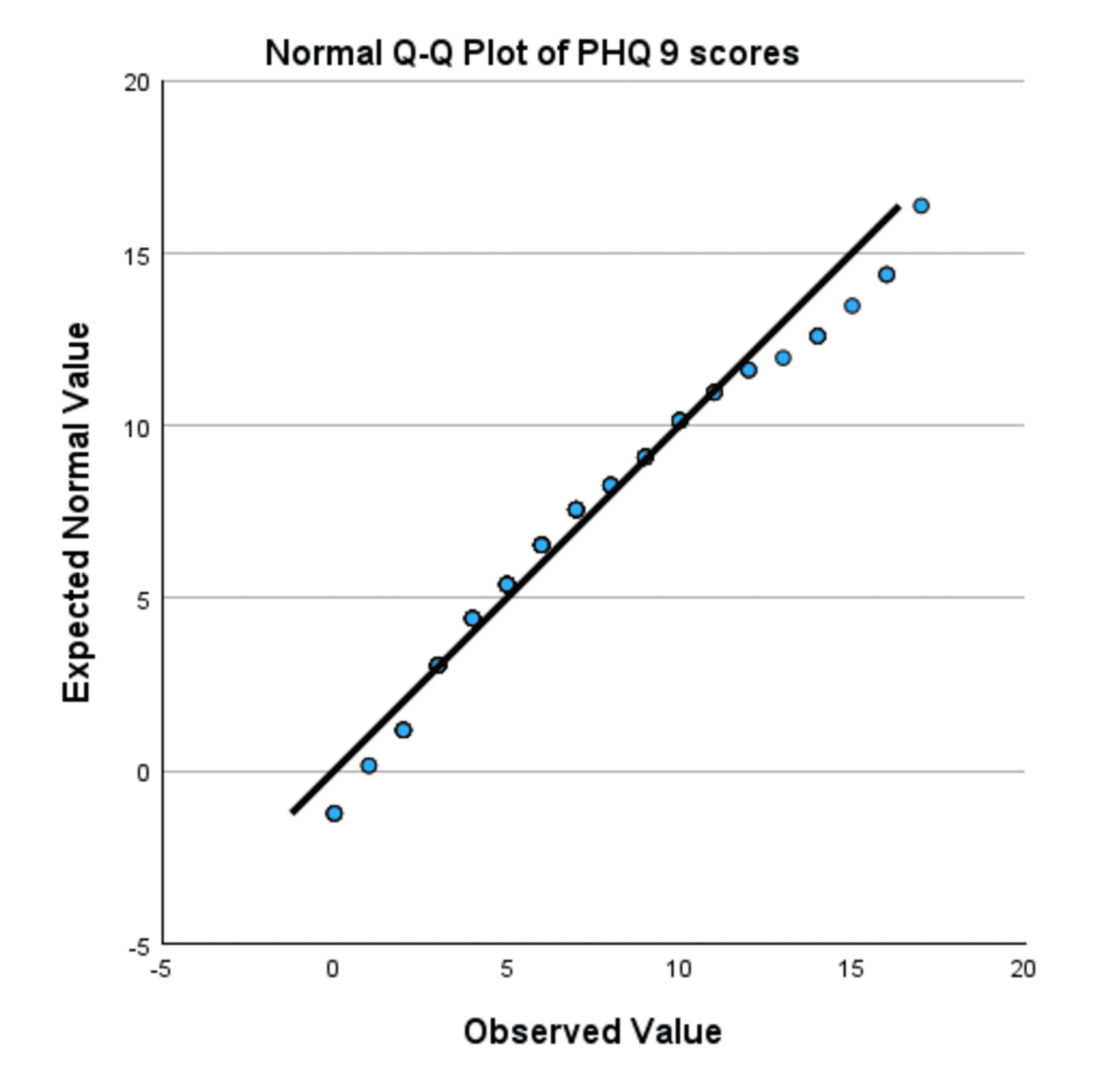
Normal Q-Q plot of the PHQ-9 scores Q-Q plot assesses whether the data is normally distributed by comparing the expected normal and observed values. PHQ-9: Patient Health Questionnaire-9, Q-Q plot: quantile-quantile plot

**Figure 3:**
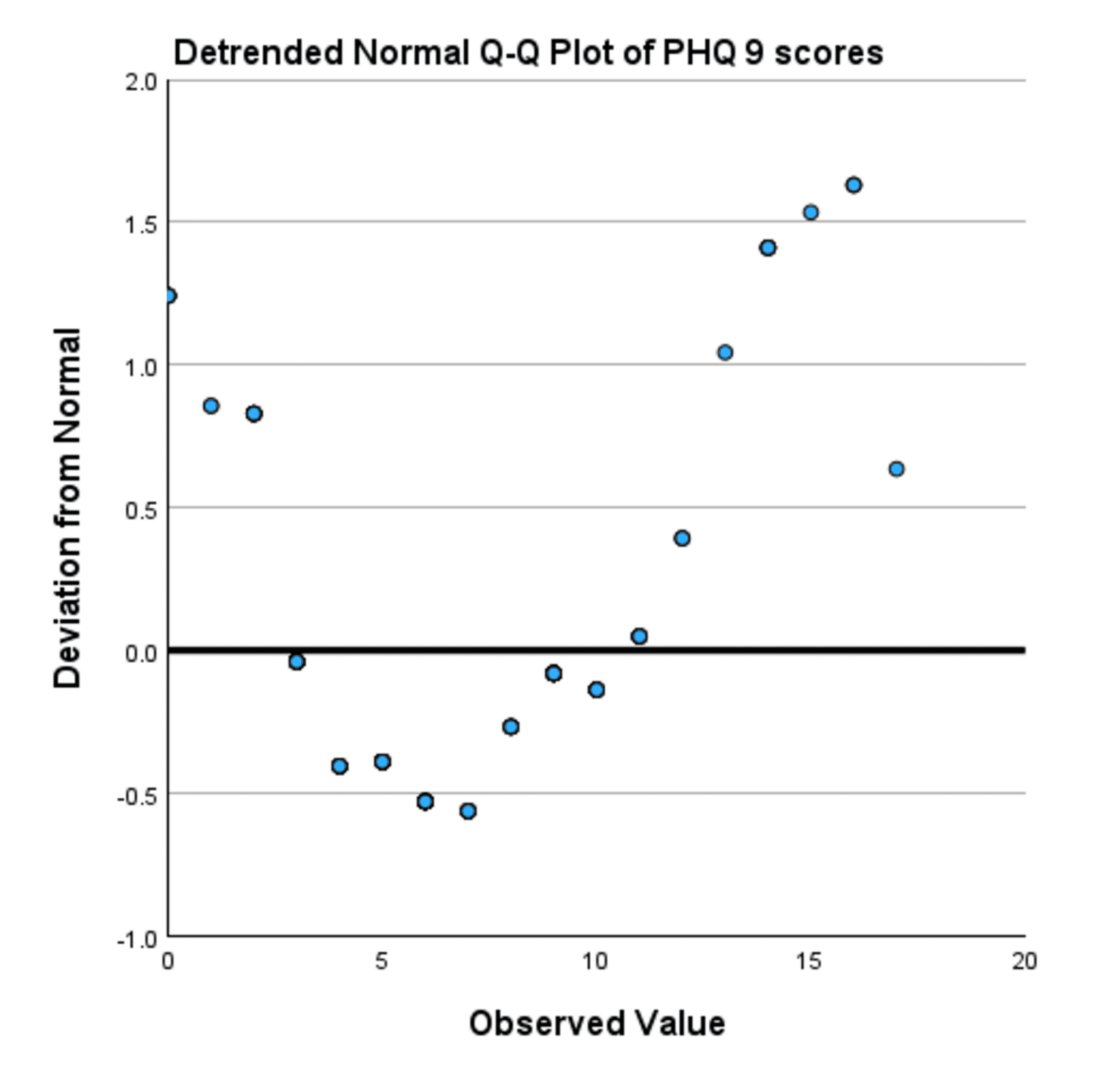
Detrended normal Q-Q plot showing most points lying close to the zero line, indicating PHQ-9 scores are fairly normally distributed in the middle range Q-Q plot assesses whether the data is normally distributed by comparing the expected normal and observed values. PHQ-9: Patient Health Questionnaire-9, Q-Q plot: quantile-quantile plot

Normality was assessed using values for skewness, kurtosis, the Kolmogorov-Smirnov test, and Q-Q plots. The skewness and kurtosis for the PHQ-9 scores for Tx-naive individuals were 0.550 and 0.151, respectively, while the skewness and kurtosis values for Tx-experienced SVR-achieved individuals were 0.723 and 0.118. The PHQ-9 had a significant Kolmogorov-Smirnov test result (D=0.1158, p<0.001). The Q-Q plot (Figure 2) and detrended Q-Q plot (Figure 3) indicated minor deviations from normality, but no extreme skewness or outliers were observed. Given these values and acceptable skewness (-1 to +1) and kurtosis (-1 to +1), the data is considered approximately normal, and parametric tests like the t-test are used.

An independent sample t-test was conducted to compare PHQ-9 scores between Tx-naive individuals and Tx-experienced SVR-achieved individuals. Levene’s test for equality of variances was nonsignificant (F=0.085, p=0.772), indicating equal variances. The t-test showed no significant difference in PHQ-9 scores between the groups, t (140)=-0.96, p=0.343, 95% CI (-0.702, 0.704). Cohen’s d effect size was 0.144, indicating a small effect, as shown in Table 3.

**Table 3:** Independent sample t-test and effect size for PHQ-9 scores between the groups Tx-naive: patients not started on DAAs and are diagnosed with chronic HCV infection. Tx-experienced SVR-achieved: patients who have achieved a 12-week course of DAAs. SVR is defined as non-detectable HCV RNA in the blood post 12- or 24-week treatment of HCV by DAAs. CI: confidence interval, df: degrees of freedom, PHQ-9: Patient Health Questionnaire-9, Tx: treatment, DAAs: direct-acting antivirals, HCV: hepatitis C virus, RNA: ribonucleic acid, SVR: sustained virologic response

Variable	Tx-naive (n=69)	Tx-experienced SVR-achieved (n=73)	T(df)	p-value	95% CI	Effect size
Mean PHQ-9 scores ± SD	6.84 ± 3.665	6.30 ± 3.818	-0.96 (140)	0.343	-0.702, 0.704	0.144

FIB-4 scores

The mean FIB-4 score in the Tx-naive group was 1.3945 with an SD of 0.74063 and a 95% CI (1.2166, 1.5724). The minimum reported score in this group was 0.34, and the maximum reported score was 4.40. Out of 69 patients, 34 (49.2%) had FIB-4 scores <1.3 and were designated as low risk of fibrosis; 32 (46.37%) had FIB-4 scores between 1.3 and 2.67 and were designated as intermediate risk of fibrosis; and three (4.2%) had a FIB-4 score >2.67 and were designated as high risk of fibrosis.

The mean FIB-4 scores in the Tx-experienced SVR-achieved group were 1.4811 with an SD of 0.82687 and a 95% CI (1.2882, 1.6740). The minimum reported score in this group was 0.36, and the maximum reported score was 4.73. Out of 73 patients, 38 (52.1%) had FIB-4 scores <1.3 and were designated as low risk of fibrosis; 30 (41.1%) had FIB-4 scores between 1.3 and 2.67 and were designated as intermediate risk of fibrosis; and five (6.85%) had FIB-4 scores >2.67 and were designated as high risk of fibrosis (Table 4).

**Table 4:** Distribution of patients based on the FIB-4 score into low, intermediate, and high risk of fibrosis FIB-4 is a non-invasive scoring system used to assess liver fibrosis, which is calculated using age, AST, ALT, and platelet count. Tx-naive: patients not started on DAAs and are diagnosed with chronic HCV infection. Tx-experienced SVR-achieved: patients who have achieved a 12-week course of DAAs. SVR is defined as non-detectable HCV RNA in the blood post 12- or 24-week treatment of HCV by DAAs. FIB-4: Fibrosis-4, AST: aspartate aminotransferase, ALT: alanine aminotransferase, Tx: treatment, DAAs: direct-acting antivirals, HCV: hepatitis C virus, RNA: ribonucleic acid, SVR: sustained virologic response

FIB-4 score	Category	Tx-naive (n=69)	% Tx-naive	Tx-experienced SVR-achieved (n=73)	% Tx-experienced SVR-achieved
<1.3	Low fibrosis risk	34	49.27	38	52.1
1.3-2.67	Intermediate fibrosis risk	32	46.37	30	41.1
>2.67	High fibrosis risk	3	4.3	5	6.85
Total		69	100	73	100

**Figure 4:**
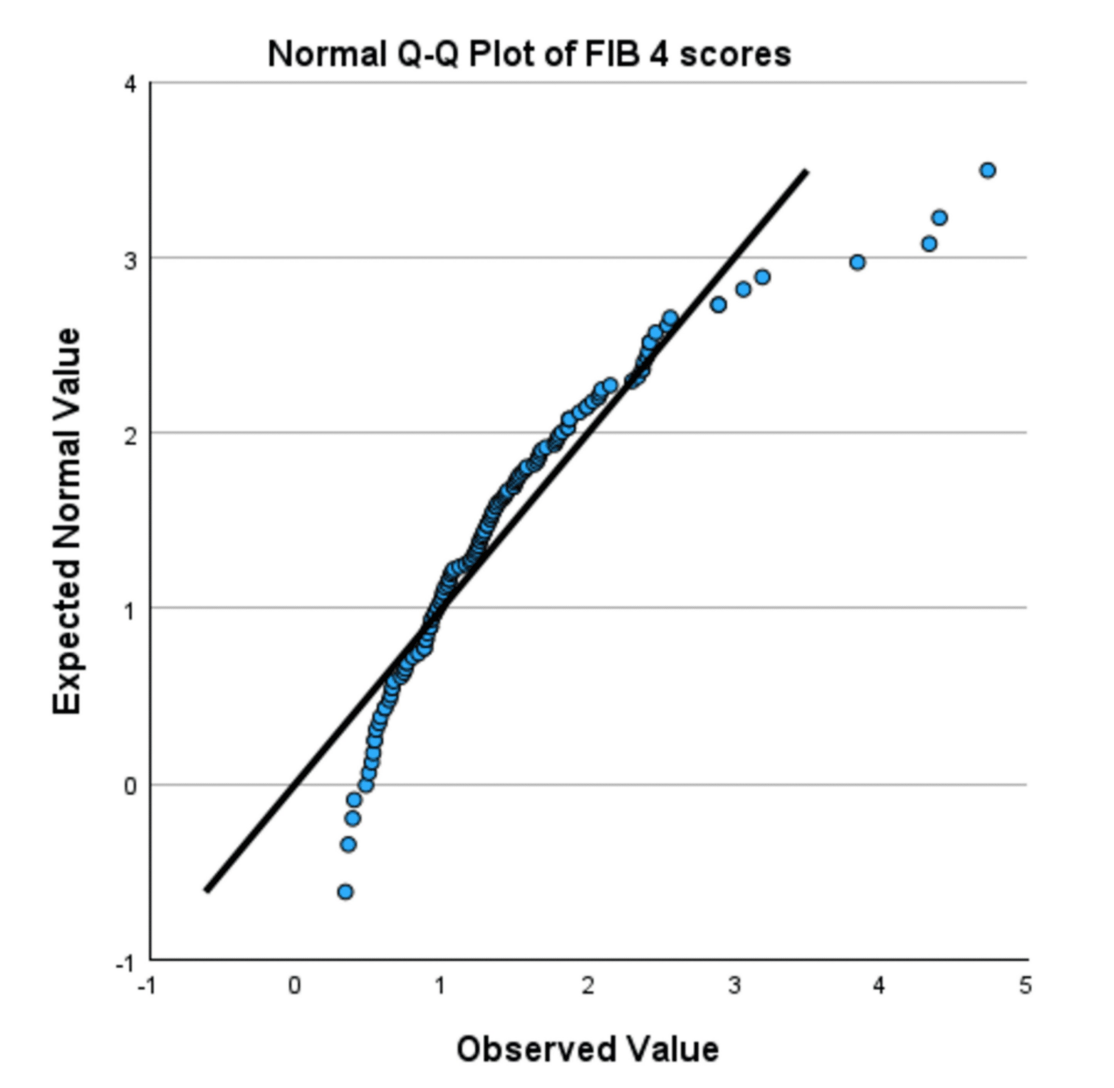
Normal Q-Q plot of the FIB-4 score distribution Q-Q plot assesses whether the data is normally distributed by comparing the expected normal and observed values. FIB-4 is a non-invasive scoring system used to assess liver fibrosis, which is calculated using age, AST, ALT, and platelet count. Q-Q plot: quantile-quantile plot, FIB-4: Fibrosis-4, AST: aspartate aminotransferase, ALT: alanine aminotransferase

**Figure 5:**
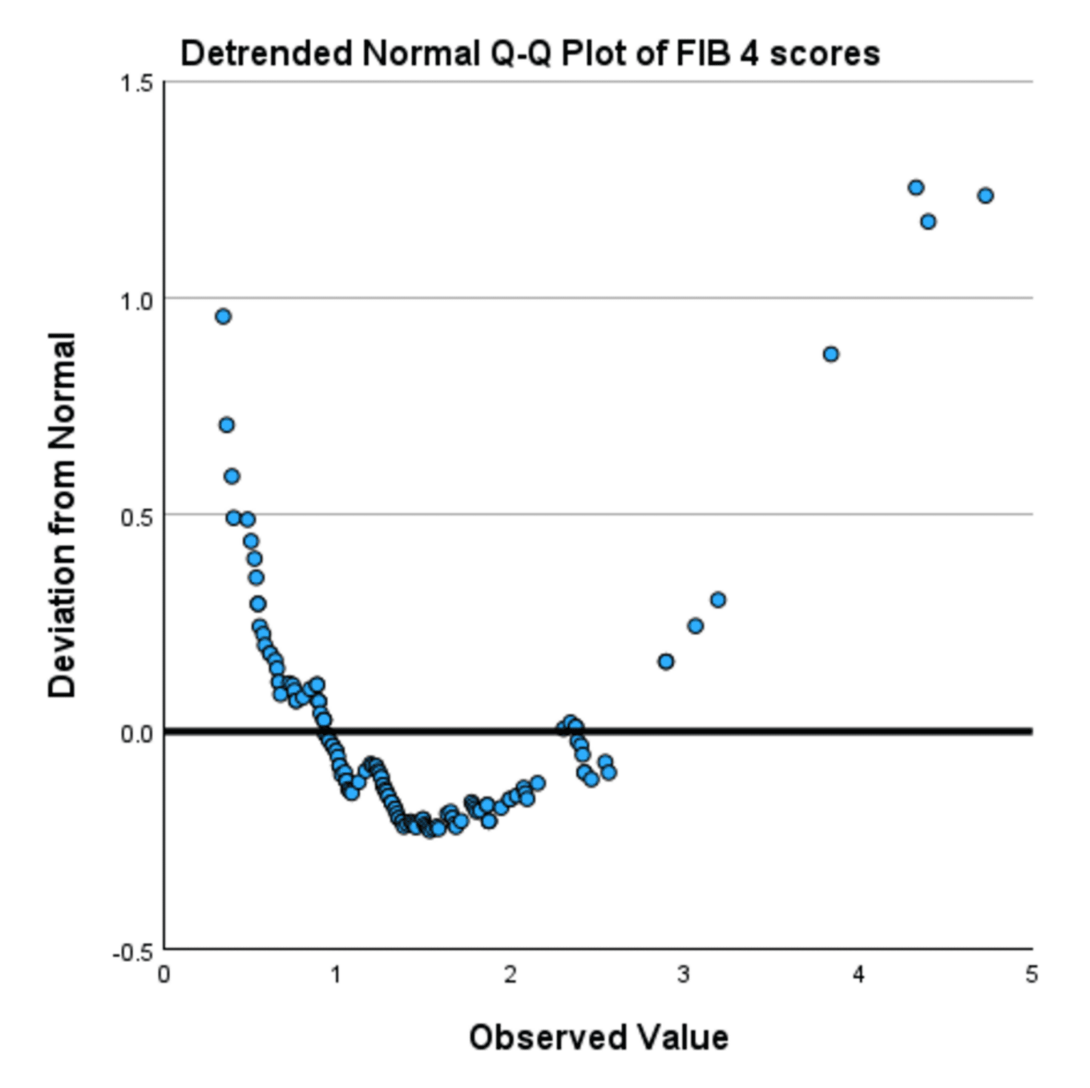
Detrended Q-Q plot showing points deviate significantly from zero both at low and high observed values suggesting non-normality Q-Q plot assesses whether the data is normally distributed by comparing the expected normal and observed values. FIB-4 is a non-invasive scoring system used to assess liver fibrosis, which is calculated using age, AST, ALT, and platelet count. Q-Q plot: quantile-quantile plot, FIB-4: Fibrosis-4, AST: aspartate aminotransferase, ALT: alanine aminotransferase

Normality was assessed using skewness, kurtosis, Q-Q plots, and the Kolmogorov-Smirnov test. The skewness and kurtosis for FIB-4 scores of Tx-naive individuals were 1.628 and 4.160, respectively, while the skewness and kurtosis for Tx-experienced SVR-achieved individuals were 1.611 and 3.702, respectively. The FIB-4 had a significant Kolmogorov-Smirnov test result (D=0.1158, p=0.0004). The Q-Q plot (Figure 4) and detrended Q-Q plot (Figure 5) showed extreme deviations from normality. This and the fact that the skewness and kurtosis lie outside the acceptable margin (+1 to -1) make the data considered as deviating from normality.

A non-parametric Mann-Whitney U test was performed to compare FIB-4 scores between Tx-naive and Tx-experienced SVR-achieved individuals. The result showed that the median FIB-4 score was not statistically significant between the two groups. The mean rank for Tx-naive individuals was 70.09, while the mean rank for Tx-experienced SVR-achieved individuals was 72.84.

The Mann-Whitney U statistic was 2421.000, Z=-0.0398, and the two-tailed p-value was 0.691. Since the p-value was greater than 0.05, there was no statistically significant difference between the FIB-4 scores between the two groups (Table 5).

**Table 5:** Mann-Whitney U test result between the FIB-4 scores of the groups FIB-4: Fibrosis-4

Test statistic	Value
Mann-Whitney U	2421.000
Wilcoxon W	4836.000
Z	-0.398
Asymp. Sig. (2-tailed)	0.691

Psychosomatic symptoms

**Figure 6:**
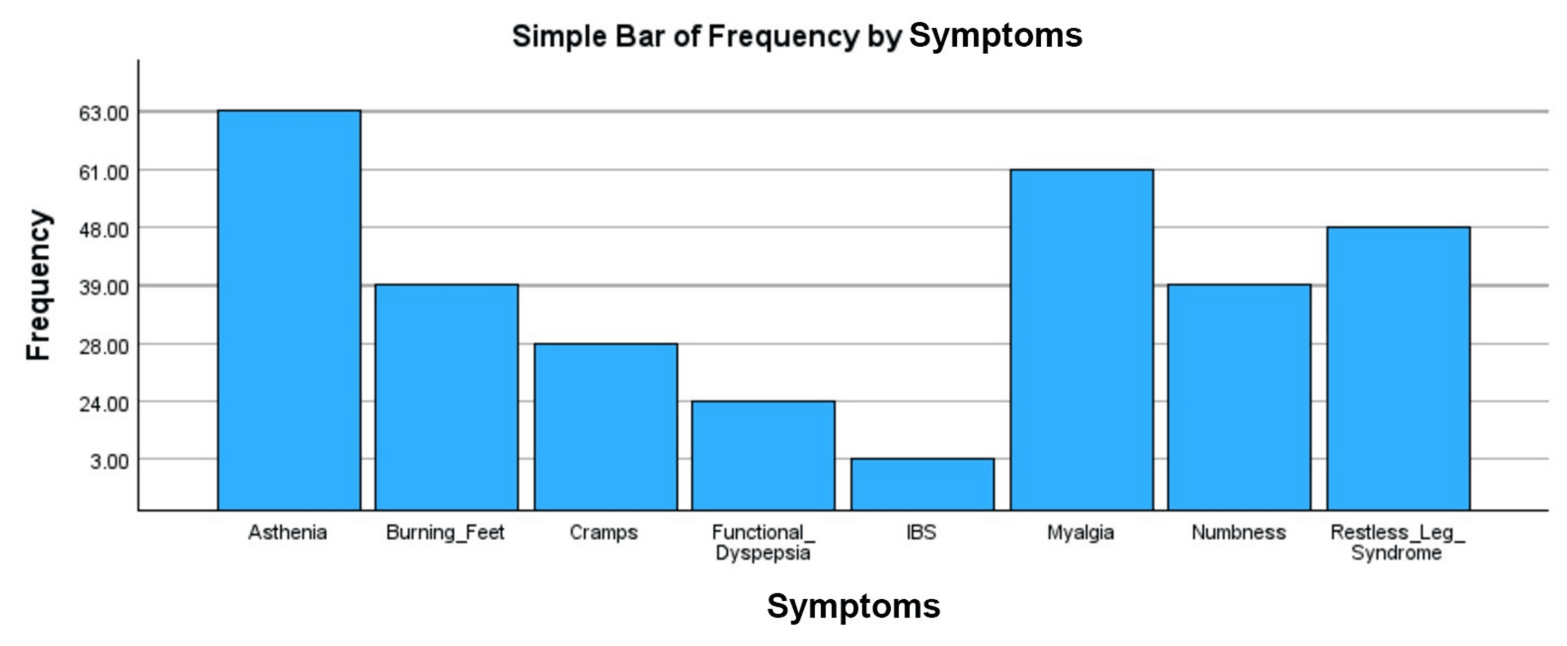
Bar chart showing the frequency of psychosomatic symptoms in Tx-naive individuals Tx-naive: patients not started on DAAs and are diagnosed with chronic HCV infection. Tx: treatment, DAAs: direct-acting antivirals, HCV: hepatitis C virus, IBS: irritable bowel syndrome

**Figure 7:**
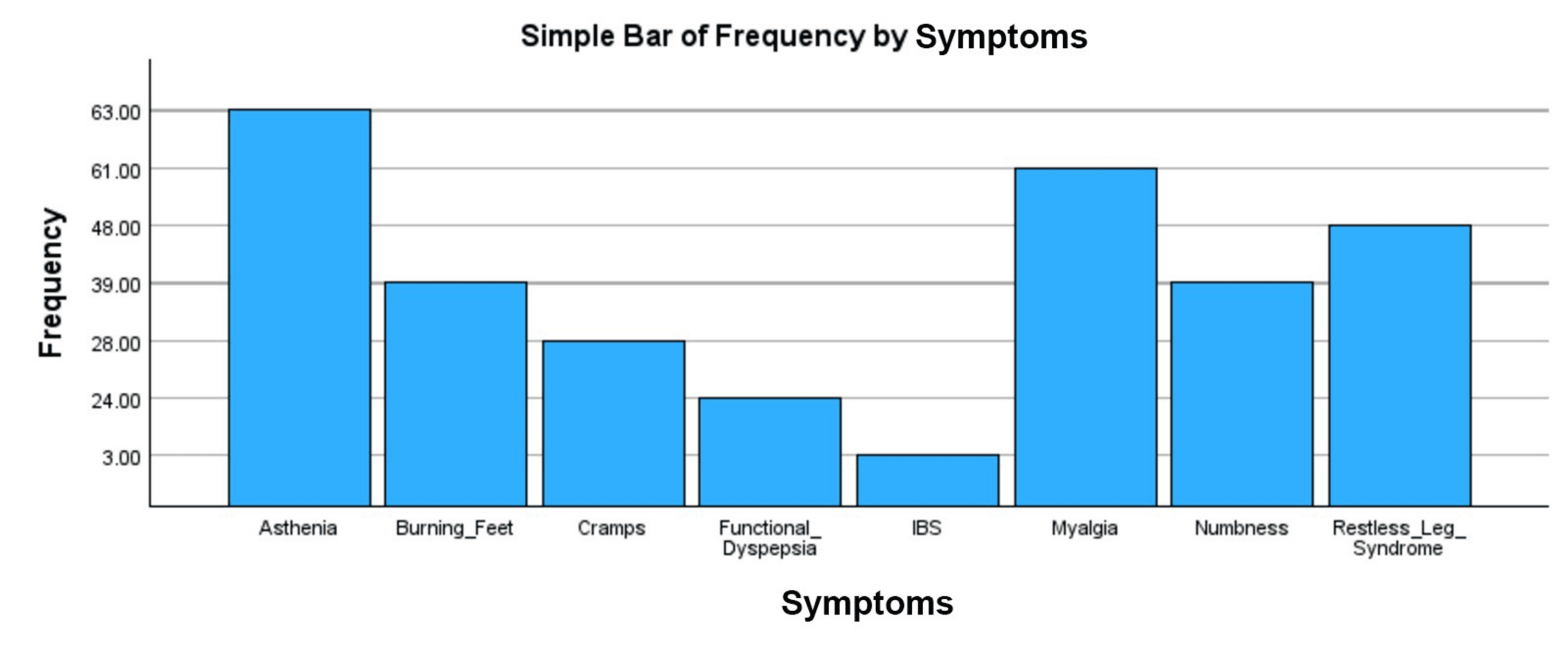
Bar chart showing the frequency of psychosomatic symptoms in Tx-experienced SVR-achieved individuals Tx-naive: patients not started on DAAs and are diagnosed with chronic HCV infection. SVR is defined as non-detectable HCV RNA in the blood post 12- or 24-week treatment of HCV by DAAs. Tx: treatment, DAAs: direct-acting antivirals, HCV: hepatitis C virus, RNA: ribonucleic acid, SVR: sustained virologic response

Prevalence of psychosomatic symptoms, i.e., burning feet, functional dyspepsia, IBS, restless leg syndrome, numbness in feet/hands, cramps, asthenia, and myalgia. Symptoms were recorded as binary responses (yes/no) as recorded as bar graphs shown in Figure 6 and Figure 7.

A Chi-squared test was performed to compare the psychosomatic symptoms between the two groups, the result of which is summarized in Table 6.

**Table 6:** Frequency of psychosomatic symptoms in Tx-naive and Tx-experienced SVR-achieved groups. The p-value is more than 0.05 showing no statistically significant difference in psychosomatic symptoms between the two groups Tx-naive: patients not started on DAAs and are diagnosed with chronic HCV infection. Tx-experienced SVR-achieved: patients who have achieved a 12-week course of DAAs. SVR is defined as non-detectable HCV RNA in the blood post 12- or 24-week treatment of HCV by DAAs. Somers'd is a measure of ordinal association that quantifies the strength and direction of the relationship between two ranked variables. df: degrees of freedom, IBS: irritable bowel syndrome, Tx: treatment, DAAs: direct-acting antivirals, HCV: hepatitis C virus, RNA: ribonucleic acid, SVR: sustained virologic response

Symptom	Tx-naive	Tx-experienced SVR-achieved	Chi-squared (df)	p-value	Somers’ d (dependent)
Burning feet	39	46	0.622(1)	0.430	0.067, SE=0.085
Functional dyspepsia	24	27	0.075(1)	0.784	0.024, SE=0.087
IBS	3	4	0.097(1)	0.756	0.060, SE=0.192
Asthenia	63	60	2.541(1)	0.111	-0.196 SE=0.116
Myalgia	61	64	1.21(1)	0.564	-0.081 SE=0.127
Restless leg syndrome	48	54	0.341(1)	0.560	0.054, SE=0.093
Cramps	28	28	0.073(1)	0.786	-0.023 SE=0.086
Numbness in feet/hands	39	47	0.918(1)	0.338	0.082, SE=0.086

The Chi-squared analysis revealed no statistically significant differences in the prevalence of psychosomatic symptoms between the Tx-naive and Tx-experienced SVR-achieved groups. All p-values exceeded the conventional significance threshold of 0.05, indicating that symptoms such as burning feet, functional dyspepsia, IBS, asthenia, myalgia, restless leg syndrome, cramps, and numbness in the extremities occurred at comparable frequencies between the two groups. The highest Chi-squared value was observed for asthenia (χ² = 2.541, p = 0.111), but this result remained nonsignificant. Additionally, as measured by Somers’ d, effect sizes were generally small, further supporting the lack of a meaningful association between treatment status and symptom prevalence. These findings suggest that achieving SVR does not appear to alter the occurrence of psychosomatic symptoms in this cohort significantly.

## Discussion

The present study aimed to evaluate the impact of HCV infection and DAA therapy on depression (PHQ-9 scores), liver fibrosis (FIB-4 scores), and psychosomatic symptoms (asthenia, myalgia, cramps, burning feet, etc.) by comparing Tx-naive and treatment-experienced SVR-achieved patients. Our findings indicate that PHQ-9 scores, FIB-4 scores, and the prevalence of psychosomatic symptoms did not differ significantly between the two groups, suggesting that HCV may be the primary driver of these symptoms rather than DAA therapy.

HCV and depression: no significant difference between groups

Our study found no statistically significant difference in PHQ-9 scores between Tx-naive and Tx-experienced SVR-achieved patients (p=0.343), with both groups exhibiting comparable levels of depression. These findings align with studies indicating that DAA therapy does not significantly influence mood disorders in HCV patients [12-15]. While some reports suggest that DAAs improve mood and quality of life following SVR [16], others indicate no effect on depression or anxiety [11,12]. Our results support the latter, suggesting that depressive symptoms observed in HCV patients are likely attributable to chronic infection, neuroinflammation, and cytokine dysregulation rather than antiviral therapy [4,5].

Fibrosis severity (FIB-4) and depression: no correlation

FIB-4 scores did not significantly differ between Tx-naive and Tx-experienced SVR-achieved individuals (p=0.691). Moreover, depression scores were not associated with liver fibrosis severity, reinforcing the hypothesis that mood disturbances in HCV are not merely a function of hepatic dysfunction but rather a result of systemic inflammation and neuroimmune activation [6]. Previous studies have reported conflicting findings regarding fibrosis and depression, with some showing a correlation [7] while others, like ours, refuting any direct association [8].

Psychosomatic symptoms in HCV: no difference post-SVR

We found no significant difference in the prevalence of asthenia, myalgia, cramps, burning feet, and other somatic symptoms between the two groups (all p>0.05). This finding contrasts with studies reporting improvement in fatigue and systemic symptoms following SVR [15,16] but aligns with those indicating that somatic complaints persist post-treatment, regardless of viral clearance [17,18]. Persistent symptoms may be due to long-term immune activation, residual inflammation, or prior neuronal damage induced by chronic HCV rather than an effect of treatment per se.

Role of socioeconomic status: a controlled variable

Unlike many prior studies, we considered socioeconomic disparities by selecting participants from a homogeneous socioeconomic background. By ensuring similar access to healthcare, education, and living conditions, we minimized the potential influence of financial stress or healthcare inequalities on depression and symptom burden. This strengthens our conclusion that HCV itself, rather than external stressors, contributes to depressive and psychosomatic symptoms.

Clinical implications

HCV infection itself, rather than DAA therapy, appears to be the major contributor to depression and somatic symptoms. Achieving SVR does not necessarily resolve mood disturbances or psychosomatic complaints, highlighting the need for long-term neuropsychiatric follow-up in HCV survivors. Routine screening for depression and somatic symptoms in HCV patients, both pre- and post-treatment, should be emphasized, as these symptoms persist despite viral clearance. Hepatitis C is increasingly recognized as a systemic disease with multiple extrahepatic manifestations, including neuropsychiatric disturbances, metabolic disorders, and immune-mediated conditions such as mixed cryoglobulinemia and lichen planus. The persistence of depressive and somatic symptoms post-SVR in our study aligns with the broader understanding that HCV can impact multiple organ systems, potentially through sustained immune activation and chronic inflammation.

Limitations and future directions

This study has some limitations. First, the cross-sectional design precludes causal inference; a longitudinal study tracking PHQ-9 and psychosomatic symptoms pre- and post-SVR would provide stronger evidence. Secondly, inflammatory cytokine levels (e.g., IL-6, TNF-α) were not measured, preventing a mechanistic analysis of persistent symptoms. Future studies should incorporate neuroinflammatory biomarkers and prospective follow-up assessments to further elucidate the underlying mechanisms.

A third limitation is the relatively low statistical power. An a priori power analysis was not conducted due to limitations in available data on expected effect sizes and variability in our study population at the time of study design. Given the study's observational nature and constraints in participant recruitment, we proceeded with a pragmatic approach, aiming to analyze all eligible patients within the study period. However, a post-hoc G*Power analysis revealed that the achieved power was 0.136 (13.6%), considerably lower than the recommended threshold of 80%. This suggests the study might be underpowered, but the findings remain meaningful, given that the results align with established literature on HCV-related neuropsychiatric and systemic symptoms. However, with a small effect size (d=0.144), the possibility of a type II error cannot be ruled out. Further studies with larger sample sizes are necessary to confirm these findings and enhance the reliability of the observed associations.

Additionally, the absence of a baseline control group without HCV infection limits the ability to compare symptom prevalence with the general population. Future studies should include a third baseline group to better determine whether neuropsychiatric and psychosomatic symptoms are directly attributable to HCV or if they reflect broader population trends.

Finally, while this study focused on evaluating depression and psychosomatic symptoms, future research should explore adjunctive psychiatric and anti-inflammatory interventions as potential strategies for improving long-term outcomes in HCV patients post-SVR. Investigating whether targeted treatments for neuroinflammation or psychiatric symptoms could modify disease-associated morbidity remains an important area for further study.

Furthermore, while this study focused on HCV, similar neuropsychiatric manifestations have been observed in other chronic viral infections, such as HIV and post-COVID-19 syndromes, which have been associated with depression, anxiety, and cognitive disturbances [21,22]. The potential role of chronic immune activation and neuroinflammation in driving these symptoms remains an area of active investigation. Future research should explore whether shared inflammatory and immune-mediated mechanisms contribute to persistent neuropsychiatric symptoms across viral illnesses and whether targeted interventions could mitigate these effects.

## Conclusions

This study suggests that HCV infection itself may play a significant role in depression and somatic symptoms rather than DAA therapy alone. While DAAs have revolutionized HCV treatment by providing a highly effective cure, their impact on mental health and systemic symptoms remains unclear. The persistence of depressive and psychosomatic complaints post-SVR challenges the assumption that viral eradication fully resolves HCV-related neuropsychiatric and systemic manifestations. These findings indicate that chronic HCV infection may have lasting effects on the central nervous system and overall physiology, potentially involving mechanisms such as neuroimmune activation, mitochondrial dysfunction, and neurotransmitter imbalances. Additionally, the lack of significant differences in FIB-4 scores between Tx-naive and Tx-experienced SVR-achieved patients suggests that these symptoms are not solely linked to liver fibrosis but could reflect broader systemic consequences of HCV.

Given that mental health disturbances and somatic complaints may persist even after viral clearance, long-term neuropsychiatric monitoring in HCV survivors is crucial. Future research should investigate the underlying biological mechanisms, incorporating neuroinflammation and autonomic dysfunction biomarkers to better understand whether these symptoms represent lingering effects of prior infection or a distinct post-viral phenomenon. Longitudinal studies tracking mental health before, during, and after DAA therapy will also help clarify the relationship between viral clearance and symptom resolution. Addressing the neuropsychiatric burden in HCV survivors remains an important clinical consideration. While DAAs have significantly improved virologic and hepatic outcomes, a comprehensive care approach integrating mental health support alongside antiviral therapy is essential for ensuring long-term well-being beyond achieving SVR.

## Figures and Tables

**Figure 8:**
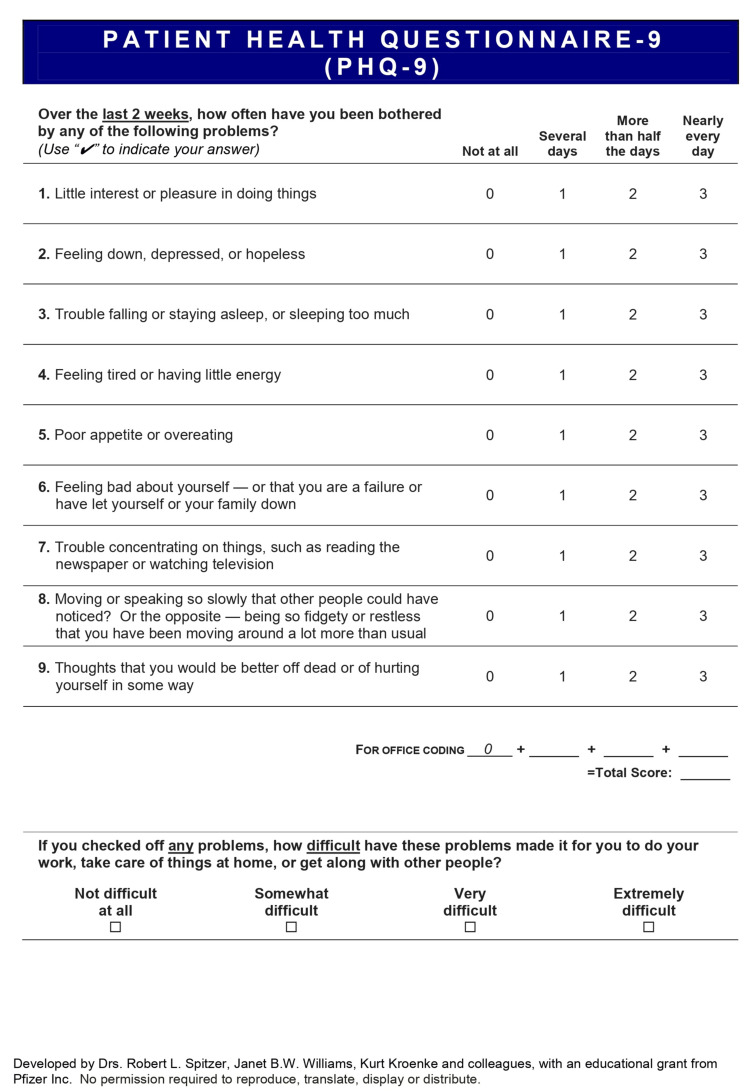
English language version of the PHQ-9 form The PHQ-9 is a validated screening tool for depression. The PHQ-9 is freely available for use and was developed by Pfizer. No special permission is required for reproduction, translation and distribution. PHQ-9: Patient Health Questionnaire-9

**Figure 9:**
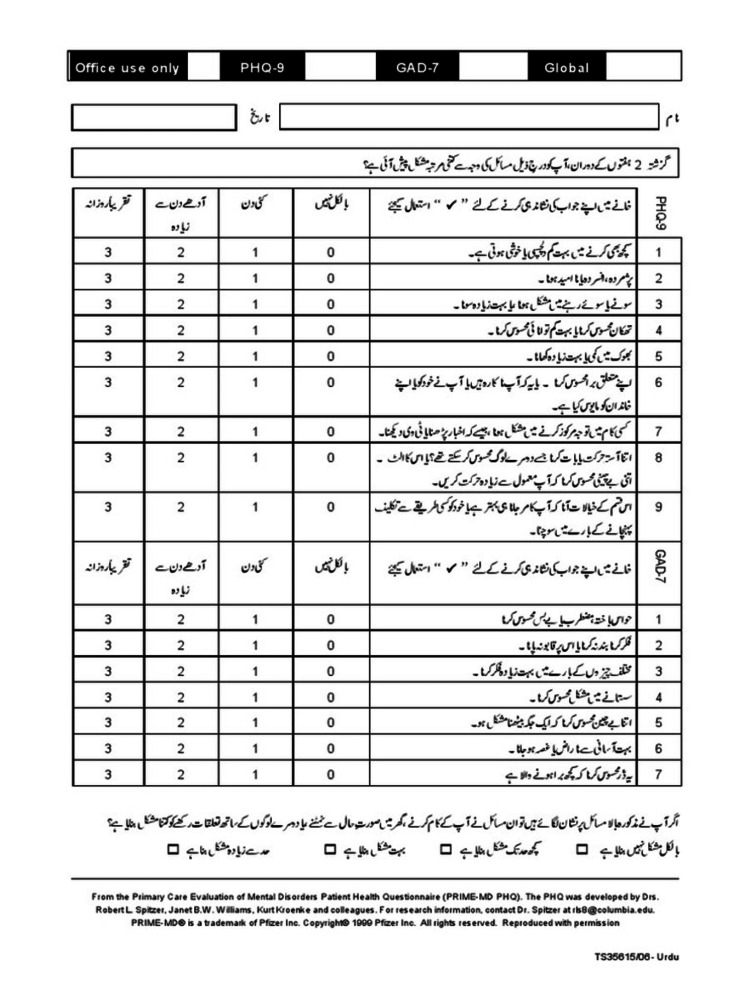
Urdu-translated version of the PHQ-9 form used in the study PHQ-9: Patient Health Questionnaire-9
